# Maria Munir Yusuf: empowering the survivors of gender-based violence

**DOI:** 10.2471/BLT.22.031222

**Published:** 2022-12-01

## Abstract

Maria Munir Yusuf talks to Gary Humphreys about the challenges faced in healing and empowering the survivors of gender-based violence in Ethiopia.

*Q: How did the idea of setting up a sanctuary for the survivors of gender-based violence come to you?*

A: It was when I was practising the law. I served on the civil and criminal bench as an examining magistrate from 1991 and saw that women lacked representation as practitioners, plaintiffs and defendants. With a group of colleagues, I set up the Ethiopian Women Lawyers Association (EWLA) in 1995 to address the problem and shortly after established myself as an independent lawyer. That brought me into contact with a lot of women who had been subjected to gender-based violence. Many had been abused by family members or were not welcome home after speaking out about their abuse. Many of them were on the street. I realized that it wasn’t enough just to provide them with legal support and started to talk about setting up a sanctuary.

*Q: Who did you talk to?*

A: Anyone who would listen. But everybody discouraged me. They asked me what I, as a lawyer, knew about running a sanctuary like that. But I felt I could do it. I had watched my mother raise 17 children, including 13 girls, and knew that a little could be made to go a very long way. I also knew what it was like to struggle without family support. After graduating high school, I fell in love with a man of a different ethnic background and religion and married him despite the objections of my family. When I was pregnant with my second son, he was arrested by the military regime and – I think – killed, although I never received official notification of his death. I am not saying that any of that was comparable to what the survivors of violence went through but I knew what it felt like to be alone and to have to fend for yourself. I also knew something about empowerment and how crucial education was in that. My father used to say to us, “You don't have any inheritance from me, but at least I can make sure you get a good education. If you are educated, you will succeed.” It was because of my education that I was able to get a job, because of my learning skills that I was able to study my way out of my predicament. In the daytime I was working for the Ministry of Labour and Social Affairs in Addis and at night I was studying for a Diploma in Law at Addis Ababa University.

*Q: When did you set up your first sanctuary?*

A: We created the Association for Women’s Sanctuary and Development (AWSAD) in 2005 and opened the first safe house for abused women, girls and children in Addis.

“We don’t just protect and heal women, we empower them.”

*Q: How did you fund it?*

A: In the beginning different friends contributed. Then we rented a house with the support of the Ark Foundation – a nongovernmental organization that provides help to needy children, college students, women and the elderly. Ark doesn’t normally work with the survivors of violence, but nobody wanted to support us, so they said, “Okay, let us start with providing you with what you need and then perhaps others will come on board.” Luckily, they did. We are still not rich, but we have many different supporters, ranging from UN agencies such as UN Women (the United Nations Entity for Gender Equality and the Empowerment of Women) to charities such as Ethiopiaid, a UK-registered charity that we formed a partnership with in 2012. We now have three safe houses in Ethiopia, in Addis Ababa, Adama and Hawassa, and have so far supported over 4500 women and girls.

*Q: Can you describe an AWSAD safe house?*

A: They generally comprise a building or group of buildings around an open area. For example, the Addis property has dormitories, classrooms, counselling rooms, a clinic, a kitchen, a playground, administrative offices and some green space. It has the capacity to serve 50, but sometimes there are as many as 100 women in residence. Even if it means putting women on a mattress on the floor, we find a way to manage.

*Q: What kind of women come to you?*

A: Around half are girls aged 11–19 and around a third of the total intake are rape victims. In most cases the rapist is known to the survivor but around four out of 10 perpetrators are strangers. Employers and employer’s relations or customers are often implicated, and it is worth noting that around a third of the survivors are housemaids. We also see many survivors of intimate partner violence and incest. I remember that the first woman who came to us when we set up in Addis was trying to protect her daughters from her husband. He had raped the first daughter and she knew that he was going to rape the second and she just left with all three. She was HIV positive and pregnant and had basically walked away from all support and shelter.

*Q: Do you offer any services to help survivors seek legal redress?*

A: Coming from my background this was very important to me. Our legal officers provide extensive support, explaining the legal process, helping survivors rehearse their testimony, and following up on their court appointments. They also accompany survivors at court hearings, appearances at the police station and the health centre for evidence collection. I should also point out that the legal service is linked to training that we give to police and prosecutors, covering topics ranging from gender-based violence survivor handling, counselling, case handling, and stress and burnout management. These capacity-building efforts have resulted in higher rates of prosecution and adjudication of cases of violence against women and girls.

*Q: How do survivors generally find you?*

A: They are generally referred to us by the police, but different women’s organizations, courts and schools also send people. Often the police are the first to become aware of the worst cases.

*Q: What services do you provide?*

A: Apart from the basics such as food and clothing and somewhere to wash and sleep, we provide a medical examination and any needed treatment. All AWSAD safe houses are equipped with licensed clinics staffed by certified nurses. An on-site health officer is also able to issue referrals when necessary. Many clients arrive at AWSAD pregnant, so the clinics provide maternal and childcare from prenatal to postnatal. As you can imagine mental health care is of enormous importance since most of the survivors arriving at the safe houses exhibit some degree of trauma. We have trained psychological counsellors who respond to each survivor’s needs, and group and individual counselling sessions are held weekly depending on consent from the resident. Where clients are suffering from extreme forms of psychological illness, we help them access psychiatric treatment at specialist hospitals. We also provide art and dance therapy. So, our safe houses are very positive happy places.

“I have seen women bounce back from the most incredible challenges.”

All of this is very important, but I want to stress that we don’t just protect and heal women, we empower them. We do this through skills development that ranges from basic literacy courses to vocational skills training. We have women and girls who come in with next to nothing and no prospects and we help them back into the world with the skills they need. These include things like food preparation, sewing, hair dressing and bamboo weaving. We also provide seed money to help women start businesses and have supported students through their studies. Several of our survivors have graduated from high school and moved on to university and careers in professions such as medicine, engineering, marketing and IT.

*Q: How long do residents typically stay?*

A: Most survivors stay a period of two months up to two years at the shelters and leave after completing skills training. We try to ensure that our exit criteria have all been met before they are helped back into the community. We use a specially developed tool to assess clients’ overall progress. Indicators such as ‘economic empowerment’, ‘health’, and ‘social empowerment’ are each scored on a scale of 1 to 10. When the criteria are met, a reintegration officer manages the exit process. When residents themselves initiate the exit process, as is often the case, assessors discuss whether they are ready to leave. I have seen women bounce back from the most incredible challenges and am often amazed by their resilience.

*Q: Can you give an example?*

A: For example, one of our survivors was repeatedly raped by her own brother and got pregnant. He organized an unsafe abortion and she haemorrhaged. When she came to us, she was really at death’s door. We made sure she got the treatment and support she needed, and she slowly got back to health, trained in hairdressing and eventually found a job. When we opened a hairdressing training salon in one of our safe houses, she came to work with us. But that is not the end of her story. She eventually got over the trauma of what she had been through and met someone and got married. We all went to the wedding, and we were all very happy for her but there was sadness too because the doctor who treated her had told her that it was impossible for her to have a child after the abortion she’d gone through. But then she became pregnant, and she delivered safely and now she’s on maternity leave. We are so happy for her. So happy. There was a lot of dancing and singing when we heard that news.

*Q: What do see for your future?*

A: I will continue to work until we get to a point where sanctuaries are not needed. Attitudes have changed in the past 25 years, but there is more work to do. When we started the EWLA, intimate partner violence was almost accepted as normal. When a man beat his wife people would say it's a sign of love. Even women would say that. And the police did not want to intervene. Now things have changed. For example, the police recognize that beating a woman is a crime, that raping a woman is a crime. We played a part in that with our capacity-building programmes for stakeholders but we have to keep working until women are safe everywhere and that is what we intend to do.

